# Amino Acid Supplementation in Fish Nutrition and Welfare

**DOI:** 10.3390/ani15111656

**Published:** 2025-06-04

**Authors:** Cláudia Aragão, Sofia Engrola, Benjamín Costas

**Affiliations:** 1 Centro de Ciências do Mar do Algarve (CCMAR/CIMAR LA), Universidade do Algarve, Campus de Gambelas, Edf. 7, 8005-139 Faro, Portugal; sengrola@ualg.pt; 2 Interdisciplinary Centre of Marine and Environmental Research (CIIMAR/CIMAR LA), Terminal de Cruzeiros do Porto de Leixões, Av. General Norton de Matos S/N, 4450-208 Matosinhos, Portugal; bcostas@ciimar.up.pt

Amino acids are not only the fundamental building blocks of proteins and, consequently, essential for animal growth, but they have also gained recognition in recent years for their critical roles in regulating key metabolic processes. In aquaculture, where the demand for more sustainable production systems continues to grow, nutrition remains a central pillar. Within this context, amino acids serve dual purposes: they are essential to balance the nutritional profiles of alternative protein sources, and they also act as functional ingredients that can enhance fish health, robustness, and resilience to stressors.

This Special Issue, “Amino Acid Supplementation in Fish Nutrition and Welfare”, brings together a collection of two review articles and six original research papers that provide up-to-date and comprehensive insights into the multifaceted roles of amino acids in fish and shrimp nutrition.

Amino acids are vital for the proper functioning of aquatic organisms, as they regulate key metabolic processes including digestion, nutrient transport, and the synthesis of enzymes and hormones. Beyond their structural role in proteins, several amino acids also contribute significantly to immune competence and stress resilience [1]. In this Special Issue, Salamanca et al. (Contribution 1) present a comprehensive review of the current literature, highlighting the physiological roles of amino acids in supporting stress mitigation and immunological responses in fish. Amino acids such as tryptophan, isoleucine, valine, arginine, and gamma-aminobutyric acid (GABA) are involved in essential physiological functions, including energy metabolism, immune regulation, and neurotransmitter activity. Additionally, phenylalanine, tyrosine, tryptophan, methionine, and taurine have been shown to modulate stress responses, enhance immune function, and reduce oxidative stress. The authors conclude that the long-term effects of amino acid supplementation remain unclear and could potentially be negative due to the risks associated with unbalanced feeding. Nevertheless, they suggest that a strategic application of amino acids before stressful events could provide tangible benefits for fish welfare.

Nile tilapia (*Oreochromis niloticus*) is one of the most widely farmed species in global aquaculture [2]. Emerging research has highlighted the critical roles of amino acids beyond protein synthesis, including the regulation of growth, reproductive performance, health status, fillet yield, and flesh quality in this species (e.g., [3,4,5]). In this context, Furuya et al. (Contribution 2) reviewed and consolidated existing data on the amino acid requirements of Nile tilapia and proposed updated recommendations. They emphasised the importance of designing requirement assays that account for amino acid interactions. Their review revealed that amino acid needs in Nile tilapia can vary significantly depending on experimental conditions such as fish strain, size, rearing system, and basal diet composition. Furthermore, amino acid recommendations may differ depending on the targeted production outcomes, such as weight gain, feed efficiency, or fillet yield. Therefore, selecting the most appropriate parameter—or combination of parameters—that aligns with the objectives of a given production system is essential. The authors also highlighted the need for more comprehensive data on amino acid requirements during the grow-out phase, as well as further validation of previously established values for key amino acids such as lysine, methionine, and threonine.

Tryptophan is an indispensable amino acid for fish, playing important roles not only in protein synthesis but also in immune tolerance mechanisms mediated by its metabolites. The main degradative pathway for tryptophan is the kynurenine pathway, which contributes to immune regulation. However, excessive activation of this pathway may lead to the accumulation of potentially cytotoxic metabolites, increasing physiological stress and suppressing immune function [6]. Vargas-Chacoff et al. (Contribution 3) evaluated the transcriptional effects of tryptophan and cortisol in primary cell cultures from the head and posterior kidney of coho salmon (*Oncorhynchus kisutch*). Their results revealed activation of the kynurenine pathway and enhanced serotonin activity following stimulation with tryptophan and cortisol, with approximately 95% of tryptophan being degraded through the kynurenine pathway. Their study emphasised the importance of understanding how this pathway is regulated and whether stressors commonly associated with aquaculture practices may trigger its activation, ultimately impacting fish health and welfare.

Considering the potential of tryptophan to modulate fish stress responses [7], Vasconcelos et al. (Contribution 4) evaluated the effects of different dietary tryptophan levels in juvenile meagre (*Argyrosomus regius*). At the end of the feeding trial, fish were exposed to a stress test consisting of 30 s of air exposure and to behaviour assessments, including anxiety-like behaviour, shoaling, and lateralisation tests. The study indicated that dietary supplementation with tryptophan, particularly at the higher inclusion level (0.8%), can reduce anxiety-like behaviour in response to acute stress (novel tank diving). Although the remaining results showed only mild effects, they provide promising evidence supporting the potential use of tryptophan as a functional dietary additive to mitigate stress in aquaculture.

The potential of dispensable (or non-essential) amino acids as functional dietary additives has received comparatively less attention in aquaculture, despite their diverse physiological roles [8]. For instance, glycine plays an important role in the synthesis of glutathione—together with glutamate and cysteine—thereby contributing to the antioxidant defence system in fish. Glycine is also known to stimulate the immune system [9]. In this context, Abbasi et al. (Contribution 5) analysed the effects of dietary glycine supplementation on the immunological and antioxidant capacities of common carp (*Cyprinus carpio*). Their findings showed that dietary glycine increased growth performance, enhanced glutathione-related antioxidant parameters and boosted humoral immune responses. Additionally, glycine supplementation improved several parameters mainly related to innate immunity in skin mucus, which may help prevent pathogen entry. Based on these results, the authors recommended a dietary supplementation level of 5 g/kg glycine for common carp feeding.

As the aquaculture industry increasingly uses alternative protein sources to replace fishmeal, methionine becomes the first limiting amino acid in many aquafeeds. This indispensable amino acid is essential for key physiological functions, including protein synthesis, detoxification, and methylation reactions [8]. Given that industrial shrimp feeds are among the largest global consumers of fishmeal, Nunes and Masagounder (Contribution 6) investigated the optimal levels of fishmeal and methionine required to maximise growth performance and economic efficiency in juvenile whiteleg shrimp (*Litopenaeus vannamei*). DL-Methionyl-DL-methionine was used as the supplemental methionine source. Their results indicate that fishmeal levels in shrimp feeds can be minimised or even eliminated without impairing growth performance, provided that dietary methionine requirements are met through effective supplementation strategies. This approach supports both improved profitability and sustainability in shrimp aquaculture.

Taurine is a sulphur-containing amino acid with important physiological functions, such as the synthesis of bile salts, which are essential for the emulsification, digestion, and absorption of dietary lipids [10,11]. The shift from fishmeal to plant-based proteins in contemporary diets may lead to taurine deficiency, as it is abundant in marine sources but virtually absent in terrestrial plants. Therefore, Aragão et al. (Contribution 7) examined the effects of taurine supplementation in low-fishmeal diets for Senegalese sole (*Solea senegalensis*). Their study showed that high inclusion levels of plant proteins negatively affected lipid metabolism, likely due to reduced bile acid synthesis and/or limited taurine availability for bile salt formation. However, taurine supplementation in these plant-based diets helped mitigate some of these adverse effects, improving lipid utilisation and supporting enhanced metabolic performance. Moreover, while taurine supplementation had a positive impact on growth, achieving this benefit appeared to require supplementation levels that exceed those typically found in conventional fishmeal-based diets.

Dietary additives hold potential for enhancing immune responses, yet limited information is available regarding their application in diets for shrimp at larval and post-larval stages. Therefore, Barreto et al. (Contribution 8) evaluated the potential beneficial effects of vitamins C and E, β-glucans, taurine, and methionine in whiteleg shrimp post-larvae. Their results showed that post-larvae fed the taurine- and methionine-supplemented diet exhibited growth performance, survival, oxidative status, and immune condition comparable to those fed a positive control diet. The antioxidant capacity and robustness were further improved when vitamin C and E levels were similar to those of the positive control. However, since the positive control diet represents a premium option, it is expected that the benefits of these additives may be greater when incorporated into more cost-effective formulations. The inclusion of β-glucans in the diets showed the most promising effects, significantly reducing lipid peroxidation. These findings suggest that tailored diets with specific health-promoting additives may help overcome key challenges in shrimp larviculture, supporting the long-term success of whiteleg shrimp farming.

This Special Issue covers a broad spectrum of topics, ranging from amino acid metabolism and nutritional requirements to the use of non-proteinogenic amino acids—such as taurine—in aquafeeds. It also examines the impacts of amino acid supplementation on stress response, immune function, antioxidant capacity, and overall health status in several aquaculture species. Furthermore, the articles highlight how specific amino acids may act as functional ingredients to enhance performance, resilience, and robustness in fish and shrimp. Collectively, these studies showcase innovative strategies and scientific advancements that are essential for advancing the sustainable development of aquaculture systems worldwide.
